# Supplementary material for: Identifying Persons with Special Healthcare Needs in Dentistry—Development and Validation of the French Case Mix Tool
Source: Int J Environ Res Public Health. 2023 Feb 8;20(4):2997. doi: 10.3390/ijerph20042997 (PMC9957372; doi:10.3390/ijerph20042997)
Supplement: Supplementary file 1 [file ijerph-20-02997-s001.zip › ijerph-2094086-supplementary.pdf]

FRENCH CASE MIX TOOL

Instructions for use:
This tool should be completed <u>at the end of each session for each patient</u>, with or without a disability. For each domain, tick the box corresponding to the patient's situation in the right-hand column (one box only per domain).

Adaptation to care	DOMAINS that may require adaptation of care to allow access to dental treatment, diagnosis or prevention	
	**COMMUNICATION**	
None	No communication problems	☐
Minor	e.g. Slow interpersonal communication; Minor leaning disability; Hearing impairment; Visual impairment; Speech or verbal communication impairment	☐
Moderate	e.g. Communication via a third person; Moderate learning disability; Deaf; Blind	☐
Major	e.g. No verbal communication; Severe learning disability; Severe dementia	☐
	**NEED FOR SEDATION, GENERAL ANAESTHESIA (GA) OR OTHER FACILITATORY TECHNIQUES**	
None	No need for facilitatory techniques for examination or treatment	☐
Minor	Need for oral premedication for examination or treatment	☐
Moderate	Need for conscious sedation or hypnosis for examination or treatment	☐
Major	Need for general anaesthesia or deep sedation with an anaesthetist for examination or treatment	☐
	**COOPERATION** during examination or treatment (with or without facilitatory techniques) (See Addendum 1*)	
None	Relaxed; Fully cooperative	☐
Minor	Uneasy; Tense; Examination or treatment possible, Patient copes despite anxiety	☐
Moderate	Reticent; Verbal opposition; Raising hands; Examination or treatment difficult	☐
Major	Patient very upset, out of contact with the reality of the threat; Examination or treatment regularly interrupted; Escape behaviour; Clinical holding/ physical restraint necessary or session abandoned	☐
	**MEDICAL HEALTH** (See Addendum 2**)	
None	Patient in good general health	☐
Minor	Patient with at least one mild or well-controlled systemic disease	☐
Moderate	Patient with at least one moderate or severe systemic disease	☐
Major	Patient with at least one severe systemic disease that is a constant threat to life	☐
	**DENTAL RISK FACTORS**	
None	No specific dental risk factor	☐
Minor	Dental risk only in relation to poor hygiene or sugary diet	☐
Moderate	Moderate dental risk related to a syndrome, dysmorphology or disease e.g. Dysphagia; Cleft lip and palate; Gastrostomy; Tracheostomy; Trismus; Spasticity	☐
Major	Combination of dental risk factors related to a syndrome, dysmorphology or disease AND related to poor hygiene or sugary diet	☐
	**AUTONOMY**	
None	Patient can access dental care without help	☐
Minor	Patient needs help to reach the dental surgery, e.g. to make an appointment; to travel to the practice (medical transport, taxi, family); wheelchair user	☐
Moderate	Patient needs to be accompanied during treatment by a carer, e.g help to transfer to dental chair; for behaviour facilitation; communication	☐
Major	Patient needs to be carried for transfers; Interruption of continuity of care due to frequent hospitalisations/acute episodes; Needs to be accompanied by several carers during treatment	☐
	**MEDICAL ADMINISTRATIVE COORDINATION** (e.g. constitution of medical history; coordination with family or professional carers, social workers; contact with legal guardians)	
None	No administrative burden	☐
Minor	Medical administrative load borne by a third person (family, social worker, institution, general medical practitioner...) or through telemedicine	☐
Moderate	Medical administrative load borne by the dentist for one sector only (medical, medico-social or medico-legal)	☐
Major	Medical administrative load borne by the dentist for and between several sectors (medical, medico-social, medico-legal)	☐

** See Modified Venham scale in Appendix 1* ***See ASA Classification in Appendix 2*

French Case Mix Tool Addendum 1:

Score	Modified Venham scale*
0	**Relaxed**: smiling, willing, able to converse, best possible working conditions. Patient displays behaviour desired by the dentist spontaneously, or as soon as asked.
1	**Uneasy**: concerned. Patient may protest briefly and rapidly to indicate discomfort. Hands remain down or partially raised to signal discomfort. The patient is willing and able to express their feelings when asked. Tense facial expression, breath occasionally held. Capable of cooperation with the dentist.
2	**Tense**: tone of voice, questions and answers reflect anxiety. During stressful procedure verbal protest, quiet crying, hands tense and raised, but not interfering much. The patient interprets the situation with reasonable accuracy and continues to cope with his/her anxiety. Protest more distracting and troublesome. The patient still complies with request to cooperate. Treatment possible.
3	**Reluctant** to accept treatment, has difficulty in correctly assessing the situational threat. Pronounced protest, crying. Uses hands to try to stop procedure. Protests out of proportion with the threat or expressed well before. Manages to cope with the situation, with great reluctance. Treatment difficult.
4	**Very disturbed** by anxiety and unable to assess the situation. General crying unrelated to treatment. Prominent body movement, sometimes requiring restraint. The patient may be accessible to verbal communication and eventually with reluctance and great effort he/she tries to control him/herself. The session is regularly interrupted by protests.
5	**Out of contact**: totally disconnected from the reality of the threat. General loud crying, struggling; inaccessible to verbal communication. Actively tries to escape. Clinical holding/ restraint essential.

**Hennequin et al. French versions of two indices of dental anxiety and patient cooperation. Eur Cells Mater, 2007; 13: 3*

French Case Mix Tool Addendum 2:

ASA PS Classification**

American Society of Anesthesiologists

Score	Definition	Adult examples, Including, but not limited to:
ASA I	A normal healthy patient	Healthy, non-smoking, no or minimal alcohol use
ASA II	A patient with mild systemic disease	Mild diseases only without substantive functional limitations. Current smoker, social alcohol drinker, pregnancy, obesity (30<BMI<40), well-controlled DM/HTN, mild lung disease
ASA III	A patient with severe systemic disease	Substantive functional limitations; One or more moderate to severe diseases. Poorly controlled DM or HTN, COPD, morbid obesity (BMI ≥40), active hepatitis, alcohol dependence or abuse, implanted pacemaker, moderate reduction of ejection fraction, ESRD undergoing regularly scheduled dialysis, history (>3 months) of MI, CVA, TIA, or CAD/stents.
ASA IV	A patient with severe systemic disease that is a constant threat to life	Recent (<3 months) MI, CVA, TIA or CAD/stents, ongoing cardiac ischemia or severe valve dysfunction, severe reduction of ejection fraction, shock, sepsis, DIC, ARD or ESRD not undergoing regularly scheduled dialysis

*** available at: www.asahq.org/standards-and-guidelines/asa-physical-status-classification-system*
